# Characteristics of chicken production systems in rural Burkina Faso: A focus on One Health related practices and food security

**DOI:** 10.1371/journal.pone.0317898

**Published:** 2025-02-03

**Authors:** Michel Dione, Guy Ilboudo, Abdoul Aziz Ouedraogo, Sidonie Aristide Ima, Brice Ouedraogo, Theodore Knight-Jones, Assèta Kagambèga, Robyn Alders

**Affiliations:** 1 International Livestock Research Institute, Health Program, Bamako, Mali; 2 International Livestock Research Institute, Health Program, Ouagadougou, Burkina Faso; 3 Ministry of Economy and Finance, National Institute of Statistics and Demography, Ouaga, Ouagadougou, Burkina Faso; 4 Institute of Environment and Agricultural Research, Department of Animal Productions, Ouagadougou, Burkina Faso; 5 International Livestock Research Institute, Health Program, Addis Ababa, Ethiopia; 6 Joseph Ki-Zerbo University, Laboratory of Molecular Biology, Epidemiology and Surveillance of Viruses and Bacteria Transmissible by Water and Food, Ouagadougou, Burkina Faso; 7 Kyeema Foundation, Brisbane City, Australia; 8 Development Policy Centre, Australian National University, Canberra, Australia; University of Ghana, GHANA

## Abstract

In addition to having cultural importance, village chicken production remains an important source of cash income for most rural households in Burkina Faso. However, strict biosecurity and good management of chicken flocks are required to reduce the risk of exposure of communities to chicken waste at household level. We characterized village production systems in rural Burkina Faso in relation to importance to food security, biosecurity, husbandry and chicken health management. We surveyed 483 chicken-producing households and carried out 20 focus group discussions separately with men and women chicken producers in Boussouma commune, a typical rural setting. Crop farming was reported as the main income-generating activity carried out by chicken producers (79.5%). Seveny six per cent of households sold chicken to local markets. Chicken production and sales were aligned to social/cultural events (religious festivals, weddings, etc…) and school-fee payment period. While men spent more revenues from chicken production on agriculture and household equipment’s, women spent more on food, education and medical expenses. The chicken management system is mainly extensive scavenging, with most farmers (81.4%) keeping 5 to 50 birds with little or no supplementary feeding and rudimentary housing. Most producers indicated that Newcastle disease was the main cause of chicken mortality. While men consider high disease burden, lack of finance, and poor chicken housing as the major constraints, women prioritized the lack of adequate chicken housing, lack of feeds and limited access to veterinary services. With locally adapted interventions that build one a One Health approach, village chicken keeping has the potential to secure and greatly improve smallholder livelihoods and household food security, while preserving public health in Burkina Faso.

## Introduction

Village chickens provide a flexible livestock production system that is widespread in most African and Asian countries [1]. Chickens often serve as a resource whose productivity can be increased with modest additional inputs [2]. In rural areas, chickens are generally owned and managed by women and children and are often essential elements of female-headed households [3,4]. By increasing the number of chickens in flocks managed by women, their ability to meet household needs will increase. They may sell chickens and/or eggs to obtain other foods, or other livestock such as goats, or use the proceeds of sale for emergencies, medicines or school fees [2].

In low-income countries such as Burkina Faso, chicken keeping is important for livelihoods for income, nutrition, and gifts to strengthen social ties [5,6]. In Burkina Faso village chickens are commonly referred to as *“poulet bicyclette”,* literally translated as *“bicycle chickens”* [7]. This is because these birds are often transported from rural to urban centers on bicycles, characteristics of most of Sub-Saharan Africa. Village chicken production has an important cultural and economic role in Burkina Faso [8], where chicken production is the second largest resource in the primary sector after agriculture, contributing 15% to the agricultural gross domestic product. It remains the primary source of cash income for rural households and thus enables them to access basic social services [9].

Given its importance, in 2016, the United Nations Development Programme Study Group recommended that policymakers extend their development efforts to emerging economic subsectors, such as livestock [10]. However, the increase in the number of chickens raised following the high demand has not been accompanied by a significant transformation of the production system. Many still do not regard village chicken farming as an important economic activity. One consequence of this is that input costs such as veterinary services, however small they may be, are difficult for farmers to accept [11].

With the exception of households supported by development programmes, within Burkina Faso there has been little change in the adoption of improved rearing techniques and infrastructure, and vaccination coverage and habitat improvement remain very limited [12].

Services to improve chicken rearing in Burkina Faso remain largely unavailable, particularly for women with limited access to inputs, extension services, and market opportunities. As a result, the offtake of smallholder chicken operations remains low due to poor husbandry and high mortality, particularly for those managed by women, as does the potential of poultry to support women’s empowerment [13]. Despite this low production, the cost‒benefit ratio of investment in village chicken production is usually favorable due to minimal input costs, resulting in the resilience of this rearing system [3,14]. Thus, an improved understanding of chicken rearing systems is needed to inform sustainable interventions to reduce chicken mortality, improve chicken health and productivity as well as human health risks associated with chickens such as food contamination and human infection from zoonotic diseases. Here, we used qualitative and quantitative approaches, to understand the key management practices of the chicken production systems in relation to food security, husbandry and health management and the constraints faced by chicken producers in a typical rural setting in Burkina Faso.

## Materials and methods

### Site selection

This study was implemented as part of formative research during the implementation of the “Poultry Losses and One Health (POLOH): Reducing losses and zoonotic risks along the poultry value chain through a One Health approach” project [15]. The overarching goal of this project is to enhance household food security and safety and improve the livelihoods of poultry smallholder producers by reducing economic losses and zoonotic risks along the value chain by developing culturally and economically appropriate, gender sensitive One Health interventions at the producer level, resulting in reduced flock mortality, zoonotic pathogen occurrence, and human health risks. This project is being implemented in one of the three provinces of Centre-Nord, namely, Sanmentenga. The Centre-Nord is the second highest poultry producing region in Burkina Faso with an estimated 3,075,696 birds of which 90% are chicken [16]. Since 2015 the Burkina Faso has been facing armed violence, especially in the northern and eastern parts of its territory, resulting in widespread displacements of its populations leaving behind their livestock. These populations are forced to settle in new areas and start up small livestock rearing such as chicken for food and income generation. In April 2021, the National Council for Relief and Emergency counted 467,738 internally displaced persons in Centre-Nord which constitutes 38.4% of all national displacements [17].

Sanmentenga province, which has 11 communes, has the highest poultry population in the region and the largest urban livestock market. The POLOH project is implemented in Boussouma commune, which has the highest poultry population density. Other criteria considered when setting the project were accessibility and security. The Boussouma commune has 60 villages with an estimated population of 106,253 inhabitants (53% women and 47% men), distributed over 17,718 households. The climate of the commune is Sudano-Sahelian, characterized by two seasons: a dry season of eight months between October and June and a rainy season of four months from June to September. During the dry season, the prevailing winds are continental trade winds or *“harmattan”* that blow over the entire municipality. Rainfall is low, irregular, with uneven spatial distribution, which significantly influences production. The average annual rainfall over the past five years varied between 600 and 1,100 mm/year [12].

**Fig 1:**
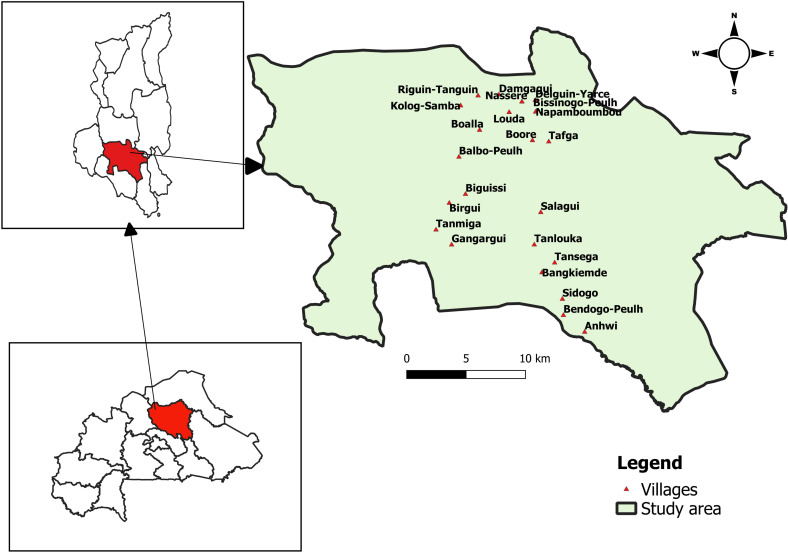
Map of Burkina Faso showing the selected region (green), commune and villages (red) (Picture credit/Guy Ilboudo).

Twenty-three villages with the highest poultry population density were purposely selected to be enrolled in the project (Fig 1).

### Sample size, data collection and analysis

#### Focus group discussions.

The Focus Group Discussions (FGDs) were carried out from February 10^th^ to February 20^th^ 2023. In each selected village, based on the census conducted in all 23 villages, we established a list of the households that kept chicken. In each of the selected villages, 10 households were selected randomly, and the household head was contacted to identify the participant. For each selected household, the participation of one man, one woman and one woman with a child under five years old was requested. Inclusion criteria for the selection of participants included being a chicken producer aged at least 18 years old and/or having good knowledge of the household’s poultry farming practices. The head of the household was therefore not necessarily a participant. The FGDs were conducted with men and women and women with a child under five years old separately making a total of three FGDs per village.

The development committee head of each village was contacted to prepare the meetings by identifying the households to be included based on availability at the time of the study and their willingness to participate. An informed consent to participate in the study was signed by all participants. During the FGDs, we developed a seasonal calendar to depict practices related to seasonality, including disease occurrence, vaccination, and flock dynamics. The discussions were facilitated using an interview guide of semi-structured questionnaire in local language (Mooré) and were recorded. Written notes were also directly taken in French by the researchers during the discussions. In total, 294 household members participated in the FGDs. This included 98 men (26 people < 35 years old and 72 people > 35 years old), 99 women (30 people < 35 years old and 69 people > 35 years old) and 99 women with a child under 5 years (66 people < 35 years old and 31 people > 35 years old).

The analysis process began with listening to the recording of the discussions and transcribing the data from local language “*Mooré*” to French. The data was examined to identify significant extracts and to identify responses according to the different pre-defined themes. We also extracted illustrative stories from chicken farmers (verbatim). After the data was processed for analysis, conclusions were drawn [18,19].

#### Household survey.

This survey was carried out from August 27^th^ to September 18^th^, 2023, as a baseline of a Randomized Controlled Trial (not reported in this paper) to assess the impacts of a set of interventions including vaccination against Newcastle disease (ND) and farmer-level behavior change communication on household/flock level characteristics. The baseline survey enabled us to document current chicken production practices, economic indicators, production indices, chicken consumption practices (meat, offals and eggs), and hygiene practices on 483 household keeping chickens across 23 villages of the project (Table 1). Sample size calculations were meant to compare households in the intervention and control arms assuming 0.5 of the control arms experienced the outcome, with 80% power, requiring 175 households in each of the intervention and control arms (95% CI if the proportion in the intervention arm is <0.35, i.e., a RR >= 1.4), requiring 350 households to be sampled by simple random sampling. However, additional households were selected to account for missing data, giving a final sample size of 483 households.

**Table 1:** List of villages and number of farmers served.

Village name	Number of households keeping chicken	Number of households sampled	% of households sampled
Bisnogo-peulh	19	2	1
Kolog-Samba	21	3	1
Anhwi	31	4	1
Balbo-peulh	39	5	1
Tafga	47	6	1
Benogo-peulh	75	9	2
Righin-tanghin	30	10	2
Tanlouka	86	11	2
Tansega	98	12	2
Boalla	108	13	3
Gangargui	123	15	3
Nassere	59	23	5
Damgagui	65	24	5
Sidogo	197	24	5
Salagui	64	25	5
Napamboumbou	70	28	6
Tamiga	91	29	6
Birgui	242	30	6
Boore	90	33	7
Bangkiemde	100	37	8
Delguin yarce	147	37	8
Louda	336	41	8
Biguissi	167	62	12
**Total**	**2305**	**483**	100

Descriptive statistical analysis was carried out using STATA/SE 17.0. These analyses included the generation of summary statistics, frequency distributions, and graphical representations to provide an overview of the data and to identify patterns and trends. For categorical variables, Chi-square tests of independence were conducted to examine associations between farmer’s demographic characteristics and farm practices. Categories with low frequencies (less than five observations) were grouped to ensure compliance with the minimum expected cell count required for the validity of the test.

For continuous variables, Student’s t-tests were employed to compare means between groups. These tests were used to assess statistically significant differences in quantitative scores, with assumptions of normality and homogeneity of variances verified prior to analysis. All statistical tests were two-sided, and a significance level of 0.05 was used to determine statistical significance.

### Ethical considerations

This study was approved by the International Livestock Research Institute Institutional Research Ethics Committee under reference: ILRI-IREC2022-57 on 10^th^ January 2023 and renewed under reference: ILRI-IREC2022-57/2 on 10^th^ June 2024. The study was also approved by the National Committee of Health Research of Burkina Faso under reference 2022-11-232 on 2^nd^ November 2022; and renewed on 5^th^ June 2024 under reference 2024-06-170.

For household surveys and FGDs the project information sheet and the consent form were read to all participants in local languages. Consenting participants were asked to sign the consent form and register their national identification card.

## Results

### Socio demographic profiles of the household heads

Table 1 shows the distribution of the households across the 23 villages surveyed.

The average years of experience of the respondents in chicken keeping was 17.9 years, with considerable variation (standard deviation = 12.6), ranging from 1 to 70 years. Mean age of participants was 49.1 (95% CI: 47.9–50.3) years, range 20 to 82 years. Only 30 respondents were women (6.2%) while 453 (93.8%) were men. Most respondents (>70%) were in age groups [35–50 years] and [50–65 years], suggesting that the sample is mainly composed of adult household heads. There was significant diversity of education levels within the sample, with most having no formal education (69.8%). Crop farming was the predominant household main economic activity (79.5%). This indicates a high economic dependence on agriculture as the main source of income. Just over one in eight (13.0%) were involved in poultry farming as a primary activity. The other main economic activities, such as other livestock farming, gold mining, and working as a small trader, represented relatively small percentages. Most respondents were married monogamously (59.8%), followed by those who married polygamously (32.7%), with widowers (5.2%) or unmarried people (1.7%). On average, polygamists have 2(CI: 1.3) wives (Table 2).

**Table 2:** Socio demographic characteristics of household heads and flock size.

Item	n	Percentage (%)
Gender		
Men	453	93.8
Women	30	6.2
Age groups of household heads		
20–34	68	14.1
35–49	191	39.5
50–64	155	32.1
65–>65	69	14.3
Level of education		
No formal education	337	69.8
Formal education (primary, secondary)	85	17.6
Adult literacy	61	12.6
Main activity		
Crop farming	384	79.5
Poultry farming	63	13.0
Other (gold mining, other livestock, trade, home gardening)	36	7.5
**Marital status**		
Married monogamy	289	59,8
Married polygamy	158	32,7
Widower	25	5,2
Single	11	1.7
Flock Size		
1–4	55	11.4
5–49	393	81.4
50–199	35	7.2
200–>200	0	0.0
**Total**	**483**	**100.0**

The mean size of the flock was 42 birds, with a minimum of 1 bird and a maximum of 155 birds, with a standard deviation of 36 birds. However, most households (81.4%) had a flock size ranging from 5 to 49 birds (Table 2).

Table 3, indicates that each household on average consists of 3 adult males including the household head, 3 adult females including the wives, 2 boys, 2 girls, 1 boys under five years old and 1 girls under five years old. For pregnant women, the average is 0.3 per household while for caregivers it is 1.

**Table 3:** Composition of the all-household members surveyed.

*Category*	*n*	*Mean*	*Std.*	*Min*	*Max*
Adult males (more than 18 years)	474	3.11	2.28	0	21
Adult females (more than 18 years)	483	3.06	2.03	1	13
Boys (6 to 18 years)	405	2.40	2.09	0	12
Girls (6 to 18 years)	407	2.42	2.27	0	22
Boys under 5 years	307	1.14	1.30	0	11
Girls under 5 years	286	1.04	1.24	0	8
Pregnant women	113	0.27	0.55	0	4
Women caregivers	251	0.75	0.89	0	5

#### Chicken entry and exit.

**Fig 2:**
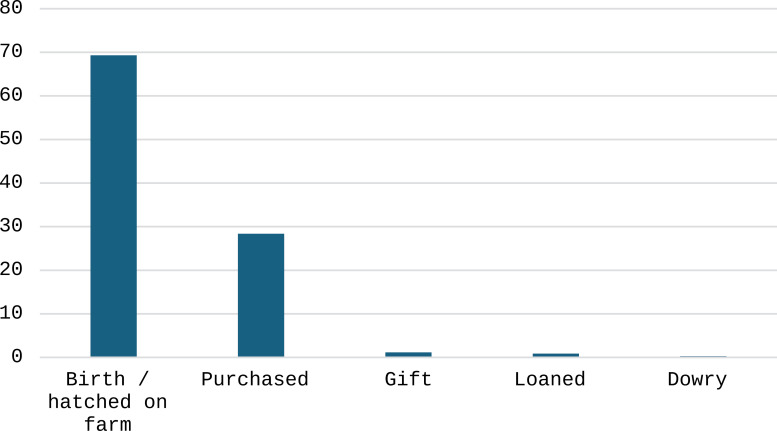
Frequency (%) of sources of chicken entry to the flock according to farmers (n = 342).

The major source of chicken entry to the holding was eggs hatched on farm, followed by birds bought (Fig 2). The chi-square test results for each category are as follows (p-values): gender (0.0014), age (0.3055), education (0.1333), farmer’s main activity (0.000), and marital Status (0.185). These results show a significant association between the sources of chicken acquisition with gender and the farmer’s main activity, suggesting that these factors influence the chickens’ entry patterns (S1 and S2 Tables).

**Fig 3:**
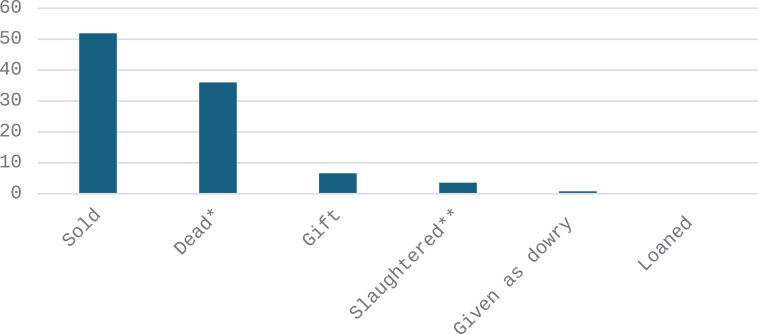
Frequency (%) of sources of chicken exit from the flock according to farmers (n = 483). *Dead following disease, accident or predation. **Slaughtered for home consumption.

The major causes of chicken exit from the flock were sales of live birds and death (Fig 3). The p-values for the Chi-square tests on the sources of exit from the flock according to different demographic categories are as follows: gender (0.952), age (0.894), education (0.291), farmer’s main activity (0.008), marital Status (0.000). These results indicate significant associations between sources of chicken exit with the farmers’ main activity and marital status (S1 and S3 Tables).

### Reasons for chicken keeping

**Fig 4:**
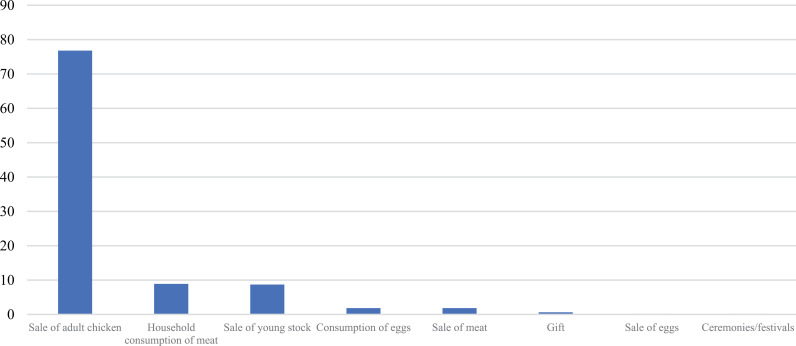
Frequency (%) in main reasons for chicken keeping (n = 483).

Seventy-six percent of producers said that the main reason for keeping chicken is for sale to generate income for the household; a few of them indicated they kept chicken for household consumption (9%), and fewer said for egg consumption (2%) (Fig 4). The p-values for the Chi-square tests on the motivations for chicken-keeping across different demographic categories are as follows: gender (0.865), age (0.154), education (0.326), farmer’s main activity (0.000), marital Status (1.000). These results reveal that there is a significant association between the reasons for chicken keeping and the farmer’s main activity (S1 and S4 Tables).

**Fig 5:**
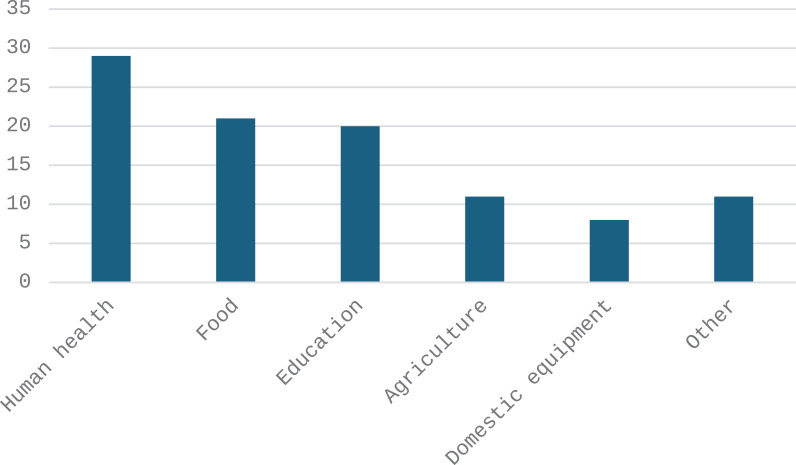
Frequency (%) in main expenditures from sales of chickens (n = 483).

Most of the revenue from sales of chicken is spent on household health (29%), food (21%) and education (20%) (Fig 5).

#### Expenses on health.

Women scored higher (3.10) than men (2.84), suggesting that they prioritize health more in their spending. The lowest average score is among 20–35-year-olds (2.62), while 35–50-year-olds had the highest score (2.95). This could reflect an increase in health care spending with age. Those who had completed adult literacy had the highest score (3.34), showing a likely correlation between awareness and health spending. People involved in gold panning (3.17) and non-poultry farming (3.00) had higher scores. Conversely, those who produce vegetables (1.50) seem to invest less in health. Divorced (3.50) and widowed (3.04) had higher scores, which may reflect specific health constraints or increased needs in these groups (S5 Table).

#### Expenses in education.

Women (2.07) scored slightly higher than men (1.97), but overall, the scores are low, showing a low relative priority for education. Score increased slightly with age up to 50–65 years old (2.02), perhaps reflect investments in education for children. Those who had completed adult literacy (2.57) allocate more expenditure to education. Small traders (2.57) and those with wage employment (2.33) had the highest scores, possibly due to stable incomes. Divorcees (2.50) and polygamous marriages (2.05) showed the highest scores (S5 Table).

#### Expenses on food.

Women (2.60) allocated more than men (2.07), often reflecting their main role in the management of food resources. The scores are generally constant, with a peak among 50–65-year-olds (2.21). The lowest scores were found among adult literates (1.92). Vegetable producers and employees had the highest scores (3.00 and 3.33 respectively), which may reflect the increased availability of resources. Single people (2.88) and widows (2.68) showed high scores (S5 Table).

#### Expenses in agriculture.

Men (1.14) allocated more than women (0.73) in agriculture. Young people aged 20–35 showed the highest scores (1.39). The scores were similar for the uneducated and the formally educated (1.15). Vegetable producers (3.00) allocated the most, showing the logic of direct investment in their activity. Cohabiting people (2.00) showed an increased interest, perhaps due to a more pronounced agricultural activity (S5 Table).

#### Expenses on home equipment.

Men (0.85) allocated more than women (0.60) in purchasing home equipment. The 20–35 age group (0.91) allocated slightly more, showing an increased need for equipment at this age. People with formal education (1.02) had the highest score. Poultry producers (1.48) invested more in household equipment. Unmarried and cohabiting people (1.00) had the highest scores (S5 Table).

#### Other expenses.

Men (1.13) allocated slightly more than women (0.90) in other expenses. The scores were broadly similar, with a slight spike among those aged 65 and over (1.25). Those with formal education (1.29) allocated more. Poultry producers (1.43) had the highest scores. Cohabiting and divorced people had the highest scores (2.00) (S5 Table).

Chicken keeping helps communities to meet their household financial needs.

This statement by a woman participant in the FGD of Bangre-kiemdé village corroborates this idea: “*With us here, it is mandatory for each head of household to raise chickens. We raise chicken because we can sell, buy food, give to a stranger or we can sell to pay for a child’s school fees, so chicken production is very beneficial for us.”*

Another woman from the same village said: *“When you can raise without them dying, you can do a lot. If you sell four to five chickens, you are rich. In addition, manure can be used as fertilizer for our fields.”*.

A man from the FGD of Damgagui village said: *“We raise chickens because it benefits us because we can sell, to pay for food, to pay our children’s school fees and to solve other problems”.*

### Chicken ownership

During the FGD, it appeared that men, women, and children could own chickens. This statement was confirmed by the household survey. However, the flock size kept by men was larger than the flock size of women and children (Table 4).

**Table 4:** Chicken ownership by different genders and age categories.

Category	n	Mean	Std.	Min	Max
Flock size	483	20.6	19.3	1	155
Owned by adult men	483	11.8	14.7	0	138
Owned by adult women	483	4.6	6.9	0	90
Owned jointly	483	1.0	4.6	0	60
Owned by children	483	3.1	6.9	0	104

The following statements during the FGDs confirm the facts:

*“(In addition to men)…The practice of chicken farming in our locality is carried out by children and women as well.”* (adult man from Nasséré’s village).

*“Whether you are a man or a woman from this village, you must have chickens. So, it makes men and women raise chickens*” (adult woman from Napambougou village).

According to a woman participant from Boré village, this situation can be explained by changes in mentality linked to the socioeconomic situation of households. She said:

*“Currently, men no longer have the means to take care of families; it is the women who take care of the families with their income from the sale of animals*.”

However, in households that are still conservative, women could raise chickens without having ownership of them, instead they use their son to bear the ownership. A woman participant from the village of Delguin Yarcé said:

*“The woman can also raise in the name of her child but not herself*.” She added: *“The head of the family can forbid you by threatening to bit you (the woman) in case your chickens are the ones that multiply the most in the household.”*

The words of a woman respondent from Salagui corroborated this idea:

*“The owner of the chickens is the head of the family. The woman buys a hen for her child, but when it produces and she wants to sell the chicks, she has to ask the head of the family.”*

#### Housing practices.

**Fig 6:**
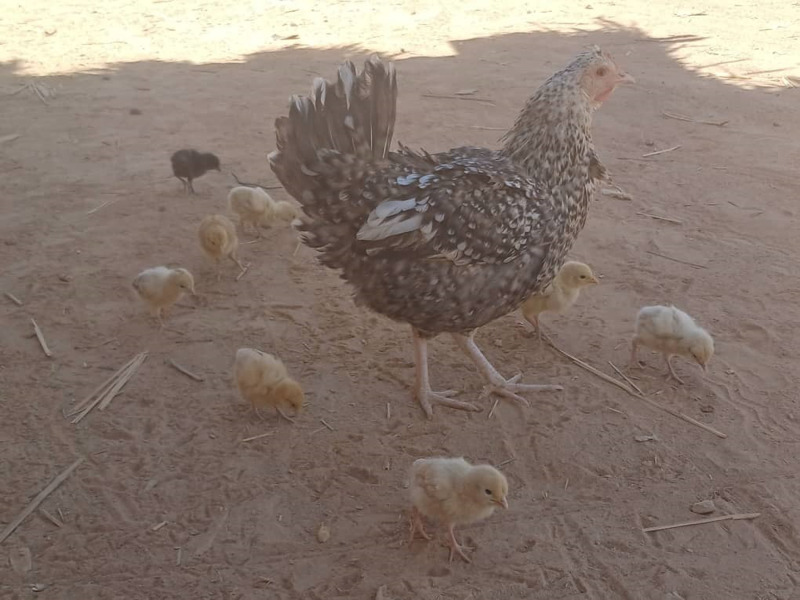
Chicken and chicks foraging for feed in Booré village, Boussouma commune, Burkina Faso (Picture credit/Michel Dione).

Both men and women reported that most producers (98%) do not confine the birds during the day (Fig 6). When the weather is hot, the birds can be located under the shade of the trees within or outside the compounds.

**Fig 7:**
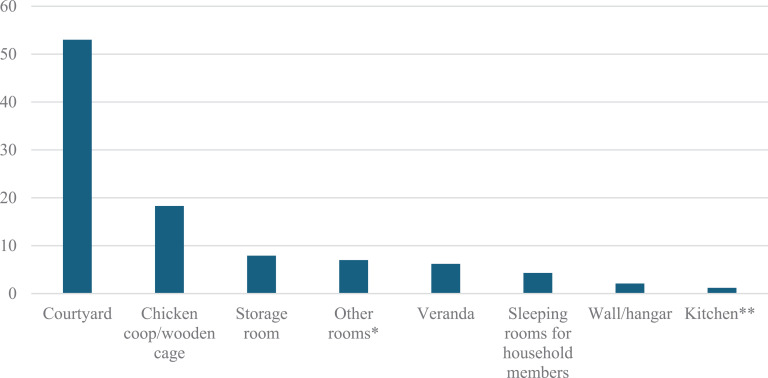
Frequency (%) in chicken confinement practices at night (n = 483). *Except storeroom and stable; **Different structure from where household members sleep.

Only 47% of farmers reported confinement of their birds at night. The housing structures include family storage room, room’s veranda, sleeping rooms and kitchen (Fig 7). Chi-2 tests for night confinement practices of chickens yield the following values for the different categories: gender (0.506), age group (0.418), education level (0.375), farmer’s main activity (0.000), marital status (0.961). Only the “ farmer’s main activity “shows a statistically significant relationship with chicken confinement at night (S6 Table).

During the FGD with men, in the village of Delguin Yarcé, a participant said:

*“The majority of farmers do not have a chicken house to shelter their chickens because they do not have the financial means. It is usually traditional equipment that is used to allow the chickens to rest*”.

A woman from Napambougou village said, *“The chickens sleep under the sheds and on the walls*.”

**Fig 8:**
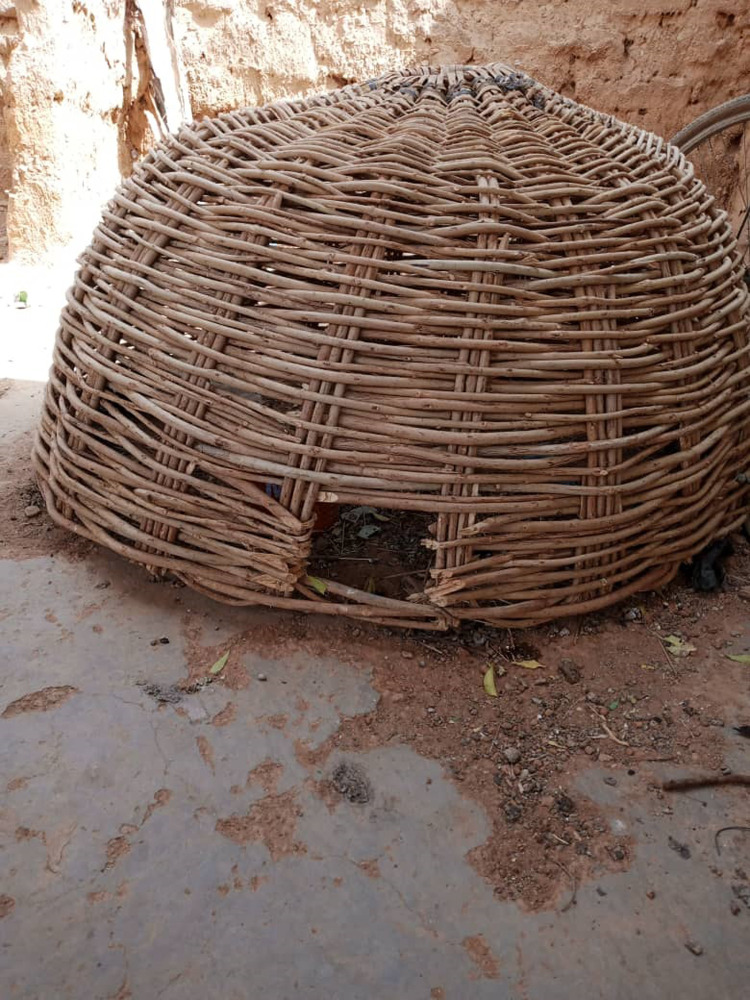
Woven basket nesting box for a hen with chicks in Booré village, Boussouma commune, Burkina Faso (Picture credit/Michel Dione).

A few farmers managed to build confinement structures using available material including stems of crops or mud bricks, while others relied on straw-woven coops, especially for laying hens (Fig 8).

According to participants in the FGDs, the lack of housing units for chickens exposes them to predators, thieves, and heat stress during harmattan season.

A woman from Salagui village stated: *“There are sparrowhawks, cats and dogs that catch them (the chickens)”*.

A man from Salagui village added*, “The lack of a chicken coop leads to high mortality of chickens in February because the chickens are exposed to the wind.”*

### Chicken genetics and reproductive practices

Ninety-nine percent (99%) of the households surveyed reported keeping local indigenous chicken ecotypes. A man from Boré village FGD said, “*We raise local chickens. It is the only breed in our village*.”

Moreover, according to the perceptions of the community, it is easier to raise local chicken breeds than exotic or “improved” breeds, referred to here as broiler chickens. Indeed, the care of local breed chickens does not require a lot of financial or labour resources compared to “improved” or broiler chickens, and these chickens are more resistant to diseases, as stipulated by one participant a woman from Boré who said, “*Local chickens are more resilient than are broilers, which is why we prefer local chickens.”*

The main method of reproduction used in the community is the traditional method, in which hens lay and incubate their fertilized eggs until the chicks’ hatch.

A woman in Nasséré village said, “*We have no other methods to get our chicks. It is the hen that lays the eggs, and covers them, until they hatch*”.

Another woman added, “*If you have the money, you can pay for eggs and add to the nest.”*

The adoption of the traditional methods for breeding and rearing chickens by both men and women is mainly due to the lack of financial resources. During the interviews, the farmers declared, *“We do not have the means, it is a bit difficult. The majority do not have a corner to put them in. It is up to everyone to find a solution to keep the chickens and their eggs safe”*.

A woman from Salagui village provided her experience by saying, “*When the hen starts laying eggs, we look for a piece of wood or iron to cover where she lays*”. Other producers set up rudimentary techniques, such as just putting pieces of bricks around to serve as a house for a laying hen.

**Fig 9:**
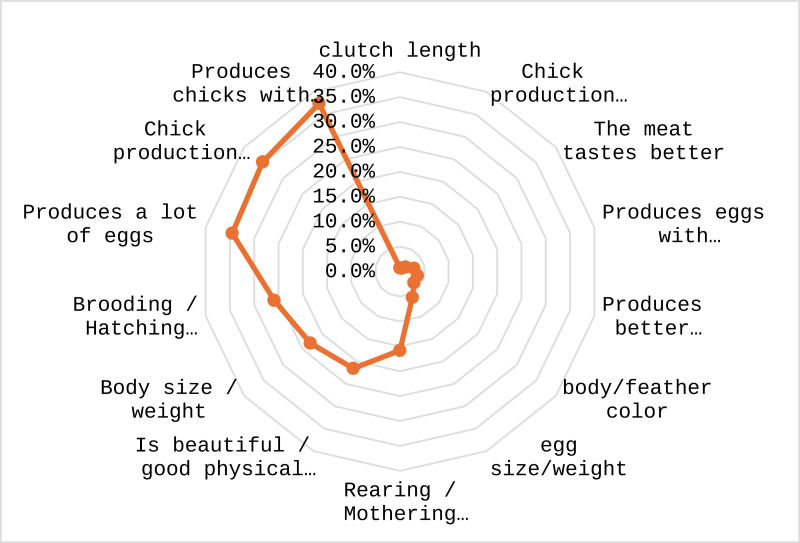
Criteria for selection of hens for breeding purpose by farmers.

Other statements explaining the non-use of “improved” breeds are the lack of financial means and infrastructure for their maintenance (feed, housing, and veterinary care). The data indicated that out of 139 respondents who engage in specific chicken selection for breeding purposes, the majority (89%) selected specific hens for breeding. In contrast, a smaller proportion (11%) reported not selecting specific hens for breeding purposes. The predominant criteria for selection of a hen by farmers include number of chicks produced, survival rate of chicks and production of eggs (Fig 9).

**Fig 10:**
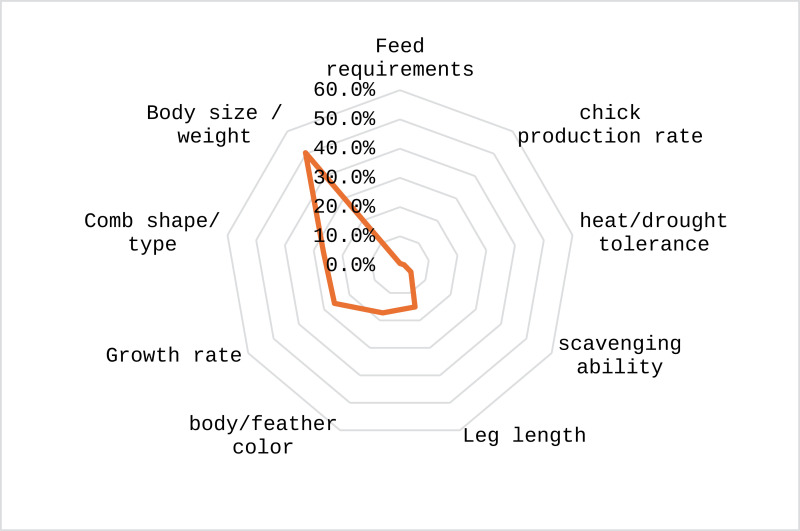
Criteria for selection of cocks for breeding purpose by farmers.

Approximately 59% of the respondents were involved in selecting cocks for breeding purposes, while about 41% of respondents reported not selecting specific cocks for breeding. The predominant criteria for selection of a cock by farmers included body weight, comb shape and growth rate (Fig 10).

### Feeding practices

**Fig 11:**
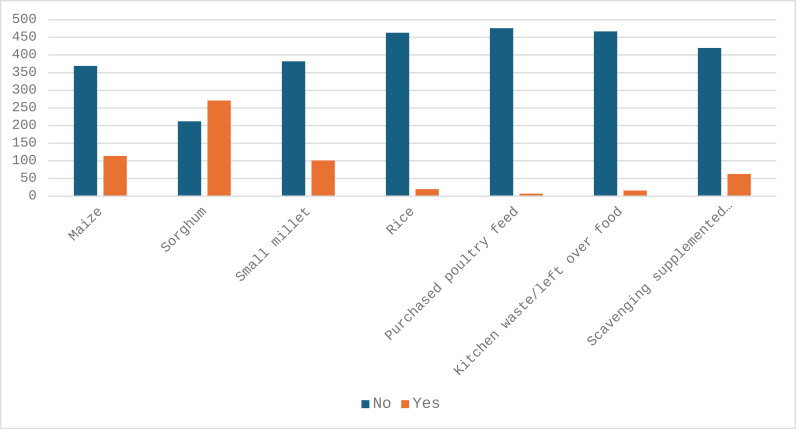
Number of farmers using types of feed available to local chickens (n = 483).

In addition to foraging, some farmers provide their chickens with supplementary feed, mainly cereals, including millet, sorghum, maize and rice, that are left over from household meals (Fig 11).

Apart from cereals, chickens also feed on termites and other insects, which they peck all day long on the ground within and outside the compound. Leftovers consumed by the farming household are also fed to chickens. Some farmers, especially women, also provide gardening products such as onion leaves, cabbage leaves, and onions during the preparation of meals. Feed supplementation with animal-source feed is not a common practice. The lack of food in the chickens’ feed is explained by the lack of financial means because, they say*, “We rarely use dried fish for the preparation of our meals, and when we do, the bones are thrown to the dog”.*

Feed is served on the ground for the chickens to peck. In the words of a male participant from Damgagui village: “*We throw the food on the ground and the water is put in the troughs; to buy feeders, we need the financial means.”.* This was echoed by a female participant from the same village who said: *“We throw the food on the ground because we do not have any equipment”.*

Ninety-three percent of the producers said they collected water from boreholes for their own use and for their chickens.

### Seasonal calendar of chicken management activities

The seasonal calendar of chicken production activities was generated by men and women during the FGDs (Table 5). The profile of the distribution of activities listed by women and men is similar, except for veterinary services. Women also reported more social events than men did. The main sociocultural events were observed in the months of October to December and in the month of February corresponding to the Christmas and New Year periods. There is more production of chickens during the months of June, July, August, September, and January, which correspond to the rainy season and prior major to social events, respectively. Chickens were mainly sold at the end and beginning of the year, i.e., December and January, corresponding to the major social events cited above. During this period, the market price of chicken was highest (up to USD 7). More feed is available toward the end of the year (October, November, and December) after the crop harvest season. This is the same time that vaccination campaigns against ND are implemented by the Government, with community animal health workers while more vaccination campaigns are implemented earlier during the rainy season (June, July, August, September). Veterinary services are available throughout the year according to men, while they are less available to the women. Vectors of disease including ticks and lice are reported to be problematic all year. The heat stress corresponding to the hotter season, called *“toulinka*” in the local language *“Mooré”*, runs from March to May prior to the peak of chicken production.

**Table 5:** Seasonal calendar of chicken production-related activities captured by men and women.

Activity	Months											
	J	F	M	A	M	J	J	A	S	O	N	D
Main social events – men	-	+ + +	-	-	-	-	-	-	-	+ + +	+ + +	+ + +
Main social events - women	+ + +	+	+	++	-	+	+			+++	+++	+++
Chicken raising	+ +	+	+	+	+	+ + +	+ + +	+ + +	+ + +	+	+	+ +
Sale of live chicken	+ + +	+	+	+	+	+	+	+	+	+	+	+ ++
Purchase of chicken replacement stock	+	+	+	+	+	+ + +	+ + +	+ + +	+ + +	+	+	+
Availability of feeds	+ +	+	+	+	+	+	+	+	+	+ + +	+ + +	+ ++
Availability of water	+ +	+	+	+	+	+ +	+ + +	+ + +	+ + +	+ +	+ +	+
Availability of veterinary services (Community Animal Health Workers -CAHWs and drugs) - men	+ +	+ + +	+ +	+ +	+ +	+ +	+ +	+ +	+ +	+ +	+ +	+ +
Availability of veterinary services (CAHWs and drugs) - women	+ +	+ + +	-	-	-	-	-	-	-	-	-	-
Vaccination campaigns	+	+	+	+	+	+	+	+	+	+++	+++	+++
Disease vectors (ticks, lice)	+ + +	+ + +	+ + +	+ + +	+ + +	+ + +	+ +	+ + +	+ + +	+ + +	+ + +	+ + +
Heat stress	–	+	+ +	+ + +	+ ++	+	+	+	+	–	–	–

+++ High; ++ Medium; + Low; - None; CAHWs, Community Animal Health Workers (in this case, village vaccinators).

### Seasonal calendar of occurrence of poultry health-related events

**Fig 12:**
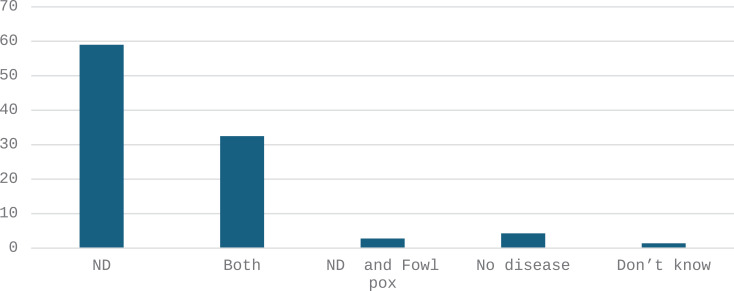
Suspected diseases that have affected chickens in the past 3 months prior to the survey according to the farmers (%, n = 483).

Fifty-nine per cent (59%) of farmers said their birds had been affected by disease compatible with ND in the 3 months prior to the survey; while 32.5% of farmers said that their birds had been affected by both suspected ND and Fowlpox (Fig 12). The chi-square test scores for each variable are as follows: gender (0.723), age group (0.372), education level (0.623), farmers main activity (0.434), marital status (0.896). None of the p-values are below the common significance level of 0.05. This means that there is no statistically significant association between categories (sex, age, education level, farmer’s main activity, marital status) and suspected diseases in chickens in the three months prior to the survey (S7 Table).

During the FGDs, men and women listed the top five suspected diseases and vectors that attack their birds: ND, Fowl pox, ticks, avian influenza and “*Nonsambga”* in “*Mooré”* (name unknown in French and English) (Table 6). The clinical signs of each disease were described by both men and women. The most economically important disease mentioned by men and women is a condition believed to be ND, having compatible clinical signs.

**Table 6:** Capturing data on chicken diseases provided by men and women.

Scientific disease name*	Newcastle disease	Fowlpox	Ticks	Unknown***	Unknown
Local name	*“Nongoume”*	*“Nonzoagma”*	*“Nonsili”*	*“Nonzouré”*	*“Nonsambga”*
Rank by economic importance by men	1^st^	2^nd^	3^rd^	4^th^	5^th^
Rank by economic importance by women	1^st^	4^th^	2^nd^	3^rd^	5^th^
Clinical signs	Mouth discharge, white diarrhea, cough, inappetence, dry paw, stiff neck	Inappetence, buttons on the beak and nose, blindness, swelling of the eyes	Presence of ticks all over the body, difficulty walking, anemia	Whitish diarrhea, weakening, buttons on the tail, sudden death	Yellowish diarrhea, vomiting, weakening, dry comb, low mortality
Age group affected	All ages	Chicks	All ages	Adults	Young
Origin of disease	Wind, consumption of meat of dead chicken	Garbage, water, feeds	Garbage	Chicken	Garbage, Water
Seasonality of the disease	December to February (cold season)	July to September (Rainy season)	All year round	No specific season	No specific season
Disease treatment methods	Traditional (not specified)	Leaves of *“Caϊcédrat”* **	Ash	Leave for threes + pepper	Traditional (not specified)
Disease prevention methods	Vaccination	Vaccination	Hygiene	No vaccine available	Leaves of *“Caϊcédrat”*
Main effect of disease on chicken	Weigh loss; Death; Aftermath	Weigh loss; Death	Weigh loss; Death	Death	Death

*This is a tentative diagnosis given that we did not perform laboratory testing. It is based on the experience of farmers, their knowledge of their systems and their interactions with animal health workers.

**Botanical name is *Khaya senegalensis.* It is a large tree that can grow up to 30 to 35 meters tall, with a short, stocky trunk that can be up to 2 meters in diameter. Their bark is gray and smooth in the first few years and then becomes flaky.

***Although the local name *“Nonzouré”* refers to high pathogenicity avian influenza (HPAI) like disease, the authors were not aware of any confirmed case of HPAI in chicken in this area.

Women recognize that different age groups of chickens are affected by these diseases as well as humans, but they have nuanced implications for fowl pox (chicks are affected) and “*Nonsambga”* (young chickens are affected). While all women reported that these diseases affect local breeds, “improved” breeds or both, not all men have knowledge of the impacts of diseases on these two types of chickens. They stated that the origin of avian diseases or the mode of contamination was due to wind, the interaction of birds with humans, free roaming, watering, people eating dead chickens, garbage, and lack of hygiene.

All men and women believe that these diseases cannot be transmitted to humans except through tick bites. They also recognize that ancestors and spirits cannot cause these diseases. Men and women use different treatments for their chickens, including vaccinations and traditional treatments (wood ash, chili pepper, tree peel, potash and shea butter). For men, preventive measures include vaccinations carried out by CAHWs and hygiene; women reinforce these measures through hygiene practices in the chicken house, sprinkling wood ash on the ground and walls of a chicken house, mixing tree leaves such as *Khaya senegalensis* in drinking water, and using a bush plant called *“Nonzoagma”* in *“Mooré”.* Despite treatments and preventive measures, these diseases kill a significant number of chickens every year. Thirty-six per cent (36%) of farmers reported death as an important route of exit from their flock (Fig 5). To address such situations, farmers expressed their will to have at least one veterinarian or CAHW in each village. They also wanted vaccination campaigns to be better organized to reach out many farmers. Others mentioned that they want veterinarians or CAHWs to go door-to-door to vaccinate chickens.

### Constraints on chicken production

While men considered the high burden of diseases, lack of finance and poor housing as major constraints to chicken farming; women considered poor or lack of housing, lack of feeds and limited access to veterinary services. Other constraints cited by men and women include the poor knowledge of producers about good practices, lack of equipment for chicken farming. The limited access to veterinary services was associated with the nonavailability of veterinary workers, and limited access to veterinary drugs.

The statement below refers to challenges related to vaccination by women:

A woman from Damgagui village said: *“Our wish is for the vaccine to be close to us in our village; we want to have a vaccinator in our village; and check we have the right equipment for vaccination”.*

Another woman participant from Delguin Yarcé added, *“All this (the challenges) sometimes leads some women into situations of sadness, discouragement, or even depression.”*

## Discussion

Poultry production is an important way of enhancing the livelihoods of rural populations, especially in low- and middle-income countries (LMICs) [14,20].

In rural Burkina Faso, village chickens play a major role as a source of income for families through the sale of live birds, and to a lesser extent the sale of their eggs. This has been reported across Africa [21]. In our study, a few farmers or their families consumed the meat or eggs of their birds; instead, the majority sold the live birds and left the eggs to hatch to increase the flock size. The profitability of village chicken production and its potential for combating hunger and malnutrition has been recognized globally by many scholars and policy makers [22,23]. But on the other hand, by generating income, communities manage to meet other important family needs, such as school fees, family health care, purchase of food, and social events. Our study show that women spend their income generated from the flock more in health, education and food, while men spend more in agriculture and house equipment. This reveals the important role of women in sustaining household food security, education and health.

Regardless of age and gender, anyone could own chickens in the participating communities. The reasons for this were highlighted during the FGDs. In the traditional *”Moaga”* society, women were not allowed to raise chickens because the house “did not belong to them”. This was mainly due to negative perceptions of men should the women’s flock out-perform theirs leading to a loss of their authority in the home. This strategy of domination has been imposed on women in West Africa mainly since the 19^th^ century [24]. As time passes, household needs, such as children’s schooling, health issues, and food consumption, increase. This means that the head of the male household would not be able to generate enough income alone, leading to realization of the importance of joint efforts for income generation, including chicken-keeping, for the security and sustainability of the household. In the end, restrictive and old perceptions have given way to the concerted management of households. This could be attributed to emancipation of women in rural communities linked to urbanization, education and change in household needs in terms of consumption and income-demands.

The production system described can be classified as being mainly extensive scavenging according to the classification of Alders, Dumas [23]. Most farmers kept between 5 and 49 birds in mixed livestock and crop systems, keeping mainly local breeds with minimum or no supplementary feeding under rudimentary housing. In this system, birds are mainly sold at local markets to generate household income, with limited household consumption of chicken eggs and meat. Chicken production and sales are aligned to social events and the school calendar during the year. This agrees with the study by Pindé, Tapsoba [25] on the characterization and typology of poultry systems in Burkina Faso, in which their results indicated that the extensive scavenging system was the most widely practiced, as it is the case in many other rural areas in the tropics [26].

The productivity of chickens is affected by the production system, which also affects the feed availability and healthcare received by the chickens [27]. Within this setting, farmers are confronted with several technical, feed and health constraints. These constraints may have negatively affected the performance of the birds, hence lowering the income of farmers.

Productive poultry necessitates meeting their nutrient requirements. Poultry require at least 38 nutrients in their diets in appropriate concentrations and balance [28]. Good nutrition is essential for flock health, survival, and productivity. The feeding system observed here is based on scavenging and supplementation with “small grains” such as sorghum (*Sorghum bicolor*), finger millet (*Eleusine coracana*), and pearl millet (*Pennisetum glaucum*). Small grains are widely used across the globe for human food and livestock feed [29] due to their drought resilience and adaptability to climate change.

These grains play vital multipurpose functions, providing nutrition to humans and as a basic chicken feed. Due to their tolerance to harsh climatic conditions, they often thrive well in marginal rainfall areas and soils with poor fertility. Moreover, they are rich in essential nutrients such as carbohydrates, zinc, calcium, iron, and phosphorus. This makes them a good feed for chicken growth and production [29]. However, due to low yields in this type of setting, feeding is inadequate contributing to low chicken productivity [30,31]. However, these communities face high poverty with fragile production systems due to recurrent drought and insecurity. This results in the limited quantity of small grains produced being used for human nutrition, rather than as chicken feed, jeopardizing the nutritional requirements of chickens. Thus, the quantity of grains served to the birds is dependent on the nutritional security of the household. Hence, it would be better to identify chicken feed sources that are not routinely consumed by human. In this regard, fresh green vegetation and insects can be used as a high-quality source of nutrition during certain periods of the year.

Our study shows that natural breeding and brooding were mainly practiced, with inadequate equipment. The use of even simple incubators has difficulties under village conditions as farmers become responsible for providing shelter, warmth and feed for the young chicks for several weeks. For reproduction, farmers select the hens or cocks based on specific criteria including weight gain, survival rate of chicks and production of eggs. These criteria align with the objectives of chicken rearing, i.e., sale of birds to generate income. During FGDs farmers highlighted their preference for local chicken ecotypes because of their resilience to the harsh weather.

Poultry populations in Africa are mostly composed of local or indigenous chicken ecotypes, appreciated for the taste and texture of their meat [27]. The introduction of exotic cocks for crossbreeding with local hens has been promoted for several years as the only alternative to improve the productivity of indigenous or local chickens. However, the genetic potential of these birds in rural settings has not been fully exploited due to the extensive and inappropriate production system (scavenging production system), in which exotic breeds often perform poorly [27].

Like Sonaiya and Swan [32], we found that men and women have relatively good knowledge of clinical signs of common chicken diseases. However, the underlying causes of disease cannot be known with high accuracy without laboratory diagnosis. Therefore, the diseases identified during the FGDs were based on speculation by farmers. Most farmers hold ND responsible for most mortalities. According to Ouedraogo, Bale [31], ND is one of the most common diseases in traditional poultry farming with very high mortality rates in Burkina Faso (Ouedraogo *et al*., 2015). With the increasing spread of high pathogenicity avian influenza (HPAI) [33], improved access to vaccines to control endemic diseases such as ND and diagnostic services is essential as it is impossible to distinguish between ND and HPAI on the basis of clinical signs [34].

### Limitations of the study

This study used participants’ perceptions and knowledge of the chicken production system to generate information for farm practices. Some information such as disease types and their occurrence may have benefited from an observational study to ascertain the true picture. However, this was not possible in our project. Also, some responses may have been biased due to lack of clarity of the question. To reduce these biases, we recruited enumerators from the community and trained on the questionnaire considering the local socio-cultural environment.

## Conclusions

Chickens are an important asset for rural communities in Burkina Faso. The chicken production system described in our study can be classified as mainly extensive scavenging, with most farmers keeping few birds with little to no supplementary feeding and rudimentary housing. Most producers consider ND to be the main cause of bird mortalities. Other constraints include poor housing and biosecurity and limited access of farmers to veterinary services which hinder chicken productivity.

Although village chicken farming is still seldom regarded as an important economic activity, it has the potential to secure and improve smallholder livelihoods and nutrition through increased production for sale and home consumption. Business models that enhance the access of producers to quality feed and veterinary inputs are required. Given the lack of veterinarians, more and better-supported community animal health workers should be engaged. Many animal welfare issues have been reported, mostly relating to poor housing, exposing birds to harsh weather and predators. Targeted interventions, based on community involvement and co-creation using a One Health approach, is needed to deliver sustainable improvements to chicken health, production and welfare in rural Burkina Faso.

## Supporting information

S1 Table Summary test de Chi-2 (P. Value) between socio demographics characteristics (column 1) and farm practices. (DOCX)

S2 Table Sources of entry to the flock according to farmers. (DOCX)

S3 Table Sources of exit from the flock according to farmers. (DOCX)

S4 Table Main reasons for chicken keeping. (DOCX)

S5 Table Main expenditures from sales of chickens. (DOCX)

S6 Table Chicken confinement practices at night. (DOCX)

S7 Table Suspected diseases that have affected chickens in the past 3 months prior to the survey according to the farmers. (DOCX)
