# Supplementary material for: Characteristics of chicken production systems in rural Burkina Faso: A focus on One Health related practices and food security
Source: PLoS One. 2025 Feb 3;20(2):e0317898. doi: 10.1371/journal.pone.0317898 (PMC11790147; doi:10.1371/journal.pone.0317898)
Supplement: S1 Table — (DOCX) [file pone.0317898.s001.docx]

Table S1: Summary test de Chi-2 (P. Value) between socio demographics characteristics (column 1) and farm practices.

|  | Type of chicken entry | Type of chicken exit | Main reasons for chicken keeping | Chicken confinement practices at night | Suspected diseases that have affected chickens in the past 3 months prior to the survey according to the farmers |
| --- | --- | --- | --- | --- | --- |
| Gender | 0.001* | 0.952 | 0.865 | 0.506 | 0.723 |
| Age | 0.306 | 0.894 | 0.154 | 0.418 | 0.372 |
| Education | 0.133 | 0.291 | 0.326 | 0.375 | 0.623 |
| Main activity | 0.000* | 0.008* | 0.000* | 0.000* | 0.434 |
| Marital status | 0.185 | 0.000* | 1.000 | 0.961 | 0.896 |

* Significant at 5%
