# Supplementary material for: Characteristics of chicken production systems in rural Burkina Faso: A focus on One Health related practices and food security
Source: PLoS One. 2025 Feb 3;20(2):e0317898. doi: 10.1371/journal.pone.0317898 (PMC11790147; doi:10.1371/journal.pone.0317898)
Supplement: S2 Table — (DOCX) [file pone.0317898.s002.docx]

Table S2: Sources of entry to the flock according to farmers

| **Gender** | Birth/ hatched on farm | Purchased | Obtained as dowry | Loaned | Gift | Total |
| --- | --- | --- | --- | --- | --- | --- |
| Male | 222 | 91 | 0 | 2 | 4 | 319 |
| Female | 15 | 6 | 1 | 1 | 0 | 23 |
| Total | 237 | 97 | 1 | 3 | 4 | 342 |
| [20-35[ | 38 | 13 | 0 | 0 | 1 | 52 |
| [35-50[ | 95 | 45 | 0 | 1 | 1 | 142 |
| [50-65[ | 75 | 32 | 1 | 2 | 0 | 110 |
| [65 et +[ | 29 | 7 | 0 | 0 | 2 | 38 |
| **Education** | 237 | 97 | 1 | 3 | 4 | 342 |
| No formal education | 157 | 65 | 0 | 1 | 3 | 226 |
| Formal education | 47 | 24 | 0 | 1 | 0 | 72 |
| Adult literacy | 33 | 8 | 1 | 1 | 1 | 44 |
| Total | 237 | 97 | 1 | 3 | 4 | 342 |
| Main activity | Birth/ hatched on farm | Purchased | Obtained as dowry | Loaned | Gift | Total |
| **Poultry farming** | 7 | 20 | 0 | 1 | 0 | 28 |
| Other livestock farming | 25 | 12 | 0 | 1 | 0 | 38 |
| Crop farming | 205 | 65 | 1 | 1 | 4 | 276 |
| Total | 237 | 97 | 1 | 3 | 4 | 342 |
| **Marital status** | Birth/ hatched on farm | Purchased | Obtained as dowry | Loaned | Gift | Total |
| Not married | 3 | 1 | 0 | 0 | 0 | 4 |
| Married monogamous | 148 | 60 | 0 | 2 | 2 | 212 |
| Married polygamous | 70 | 29 | 0 | 0 | 2 | 101 |
| Divorced | 0 | 1 | 0 | 0 | 0 | 1 |
| Widow | 16 | 6 | 1 | 1 | 0 | 24 |
| Total | 237 | 97 | 1 | 3 | 4 | 342 |
