# Supplementary material for: Characteristics of chicken production systems in rural Burkina Faso: A focus on One Health related practices and food security
Source: PLoS One. 2025 Feb 3;20(2):e0317898. doi: 10.1371/journal.pone.0317898 (PMC11790147; doi:10.1371/journal.pone.0317898)
Supplement: S3 Table — (DOCX) [file pone.0317898.s003.docx]

Table S3: Sources of exit from the flock according to farmers

| **Gender** | Mortality/Slaughtering | Sold | Given as dowry | Loaned | Gift | Total |
| --- | --- | --- | --- | --- | --- | --- |
| Male | 194 | 246 | 4 | 1 | 31 | 476 |
| Female | 8 | 13 | 0 | 0 | 2 | 23 |
| Total | 202 | 259 | 4 | 1 | 33 | 499 |
| **Age group** | Mortality/Slaughtering | Sold | Given as dowry | Loaned | Gift | Total |
| [20-35[ | 34 | 32 | 1 | 0 | 5 | 72 |
| [35-50[ | 84 | 114 | 2 | 0 | 13 | 213 |
| [50-65[ | 58 | 76 | 0 | 1 | 10 | 145 |
| [65 et +[ | 26 | 37 | 1 | 0 | 5 | 69 |
| Total | 202 | 259 | 4 | 1 | 33 | 499 |
| **Education** | Mortality/Slaughtering | Sold | Given as dowry | Loaned | Gift | Total |
| No formal education | 132 | 184 | 1 | 1 | 20 | 338 |
| Formal education | 42 | 37 | 1 | 0 | 6 | 86 |
| Adult literacy | 28 | 38 | 2 | 0 | 7 | 75 |
| Total | 202 | 259 | 4 | 1 | 33 | 499 |
| **Main activity** | Mortality/Slaughtering | Sold | Given as dowry | Loaned | Gift | Total |
| Poultry farming | 4 | 33 | 1 | 0 | 2 | 40 |
| Other livestock farming | 18 | 20 | 0 | 0 | 4 | 42 |
| Crop farming | 180 | 206 | 3 | 1 | 27 | 417 |
| Total | 202 | 259 | 4 | 1 | 33 | 499 |
| **Marital status** | Mortality/Slaughtering | Sold | Given as dowry | Loaned | Gift | Total |
| Not married | 1 | 3 | 0 | 0 | 0 | 4 |
| Married monogamous | 126 | 154 | 1 | 0 | 19 | 300 |
| Married polygamous | 66 | 92 | 2 | 1 | 11 | 172 |
| Concubinage | 0 | 1 | 1 | 0 | 1 | 3 |
| Divorced | 1 | 0 | 0 | 0 | 0 | 1 |
| Widow | 8 | 9 | 0 | 0 | 2 | 19 |
| Total | 202 | 259 | 4 | 1 | 33 | 499 |
