# Supplementary material for: Characteristics of chicken production systems in rural Burkina Faso: A focus on One Health related practices and food security
Source: PLoS One. 2025 Feb 3;20(2):e0317898. doi: 10.1371/journal.pone.0317898 (PMC11790147; doi:10.1371/journal.pone.0317898)
Supplement: S4 Table — (DOCX) [file pone.0317898.s004.docx]

Table S4: Main reasons for chicken keeping

| **Gender** | Live adult sales | for meat consumption | to sell their young | For meat sale | For egg consumption | For egg sale | To give away | For ceremonies / festivals | Not applicable | Total |
| --- | --- | --- | --- | --- | --- | --- | --- | --- | --- | --- |
| Male | 345 | 42 | 41 | 8 | 8 | 1 | 3 | 1 | 4 | 453 |
| Female | 26 | 1 | 1 | 1 | 1 | 0 | 0 | 0 | 0 | 30 |
| Total | 371 | 43 | 42 | 9 | 9 | 1 | 3 | 1 | 4 | 483 |
| **Age group** | Live adult sales | for meat consumption | to sell their young | For meat sale | For egg consumption | For egg sale | To give away | For ceremonies / festivals | Not applicable | Total |
| [20-35[ | 50 | 3 | 10 | 3 | 0 | 0 | 1 | 0 | 1 | 68 |
| [35-50[ | 152 | 18 | 13 | 1 | 4 | 0 | 1 | 1 | 1 | 191 |
| [50-65[ | 122 | 12 | 14 | 3 | 4 | 0 | 0 | 0 | 0 | 155 |
| [65 et +[ | 47 | 10 | 5 | 2 | 1 | 1 | 1 | 0 | 2 | 69 |
| Total | 371 | 43 | 42 | 9 | 9 | 1 | 3 | 1 | 4 | 483 |
| **Education** | Live adult sales | for meat consumption | to sell their young | For meat sale | For egg consumption | For egg sale | To give away | For ceremonies / festivals | Not applicable | Total |
| No formal education | 260 | 34 | 26 | 7 | 4 | 1 | 2 | 1 | 2 | 337 |
| Formal education | 66 | 4 | 9 | 2 | 1 | 0 | 1 | 0 | 2 | 85 |
| Adult literacy | 45 | 5 | 7 | 0 | 4 | 0 | 0 | 0 | 0 | 61 |
| Total | 371 | 43 | 42 | 9 | 9 | 1 | 3 | 1 | 4 | 483 |
| **Main nactivty** | Live adult sales | for meat consumption | to sell their young | For meat sale | For egg consumption | For egg sale | To give away | For ceremonies / festivals | Not applicable | Total |
| Poultry farming | 38 | 22 | 2 | 0 | 0 | 1 | 0 | 0 | 0 | 63 |
| Other livestock farmi | 30 | 0 | 3 | 1 | 0 | 0 | 1 | 0 | 1 | 36 |
| Crop farming | 303 | 21 | 37 | 8 | 9 | 0 | 2 | 1 | 3 | 384 |
| Total | 371 | 43 | 42 | 9 | 9 | 1 | 3 | 1 | 4 | 483 |
| **Matital status** | Live adult sales | for meat consumption | to sell their young | For meat sale | For egg consumption | For egg sale | To give away | For ceremonies / festivals | Not applicable | Total |
| Not married | 8 | 0 | 0 | 0 | 0 | 0 | 0 | 0 | 0 | 8 |
| Married monogamous | 225 | 20 | 29 | 5 | 5 | 0 | 1 | 1 | 3 | 289 |
| Married polygamous | 114 | 21 | 12 | 3 | 4 | 1 | 2 | 0 | 1 | 158 |
| Concubinage | 1 | 0 | 0 | 0 | 0 | 0 | 0 | 0 | 0 | 1 |
| Divorced | 2 | 0 | 0 | 0 | 0 | 0 | 0 | 0 | 0 | 2 |
| Widow | 21 | 2 | 1 | 1 | 0 | 0 | 0 | 0 | 0 | 25 |
| Total | 371 | 43 | 42 | 9 | 9 | 1 | 3 | 1 | 4 | 483 |
