# Supplementary material for: Characteristics of chicken production systems in rural Burkina Faso: A focus on One Health related practices and food security
Source: PLoS One. 2025 Feb 3;20(2):e0317898. doi: 10.1371/journal.pone.0317898 (PMC11790147; doi:10.1371/journal.pone.0317898)
Supplement: S5 Table — (DOCX) [file pone.0317898.s005.docx]

Table S5: Main expenditures from sales of chickens

| Category | Health | Education | Food | Agriculture | Domestic equipment | Other |
| --- | --- | --- | --- | --- | --- | --- |
| **Gender** | Score | Score | Score | Score | Score | Score |
| Male | 2,84 | 1,97 | 2,07 | 1,14 | 0,85 | 1,13 |
| Female | 3,10 | 2,07 | 2,60 | 0,73 | 0,60 | 0,90 |
| Total | 2,86 | 1,97 | 2,10 | 1,11 | 0,84 | 1,12 |
| **Age group** | Score | Score | Score | Score | Score | Score |
| [20-35[ | 2,62 | 1,85 | 2,07 | 1,39 | 0,91 | 1,15 |
| [35-50[ | 2,95 | 1,97 | 2,04 | 1,11 | 0,85 | 1,08 |
| [50-65[ | 2,86 | 2,02 | 2,21 | 0,99 | 0,82 | 1,09 |
| [65 et +[ | 2,84 | 1,98 | 2,07 | 1,10 | 0,76 | 1,25 |
| Total | 2,86 | 1,97 | 2,10 | 1,11 | 0,84 | 1,12 |
| **Education** | Score | Score | Score | Score | Score | Score |
| No formal education | 2,82 | 1,91 | 2,14 | 1,15 | 0,83 | 1,15 |
| Formal education | 2,67 | 1,78 | 2,08 | 1,15 | 1,02 | 1,29 |
| Adult literacy | 3,34 | 2,57 | 1,92 | 0,85 | 0,62 | 0,69 |
| Total | 2,86 | 1,97 | 2,10 | 1,11 | 0,84 | 1,12 |
| **Main activity** | Score | Score | Score | Score | Score | Score |
| Poultry farming | 2,33 | 1,57 | 1,79 | 1,40 | 1,48 | 1,43 |
| Other livestock farmi | 3,00 | 2,33 | 1,80 | 0,93 | 0,80 | 1,13 |
| Crop farming | 2,96 | 2,03 | 2,14 | 1,05 | 0,74 | 1,07 |
| Salaried employment | 1,33 | 2,33 | 3,33 | 1,67 | 0,33 | 1,00 |
| Small trader | 2,71 | 2,57 | 2,14 | 1,14 | 0,57 | 0,86 |
| Gold panning | 3,17 | 1,33 | 2,33 | 1,17 | 0,83 | 1,17 |
| Vegetables production | 1,50 | 1,00 | 3,00 | 3,00 | 0,75 | 0,75 |
| Other | 2,33 | 1,33 | 3,00 | 1,33 | 1,00 | 1,00 |
| Total | 2,86 | 1,97 | 2,10 | 1,11 | 0,84 | 1,12 |
| **Marital status** | Score | Score | Score | Score | Score | Score |
| Not married | 2,38 | 1,50 | 2,88 | 0,75 | 1,00 | 1,50 |
| Married monogamous | 2,85 | 1,94 | 2,07 | 1,16 | 0,85 | 1,13 |
| Married polygamous | 2,86 | 2,05 | 2,06 | 1,11 | 0,84 | 1,09 |
| Concubinage | 3,00 | 2,00 | 0,00 | 2,00 | 1,00 | 2,00 |
| Divorced | 3,50 | 2,50 | 1,00 | 0,50 | 0,50 | 2,00 |
| Widow | 3,04 | 1,92 | 2,68 | 0,72 | 0,68 | 0,96 |
| Total | 2,86 | 1,97 | 2,10 | 1,11 | 0,84 | 1,12 |
