# Supplementary material for: Characteristics of chicken production systems in rural Burkina Faso: A focus on One Health related practices and food security
Source: PLoS One. 2025 Feb 3;20(2):e0317898. doi: 10.1371/journal.pone.0317898 (PMC11790147; doi:10.1371/journal.pone.0317898)
Supplement: S6 Table — (DOCX) [file pone.0317898.s006.docx]

Table S6: Chicken confinement practices at night

| **Gender** | Inside the structure where household members sleep | Kitchen (different structure from where household members sleep) | On the veranda | In the courtyard | Storage room | Chicken coop/wooden cage | Other rooms (except storeroom and stable) | Other the wall/Hangar | Total |
| --- | --- | --- | --- | --- | --- | --- | --- | --- | --- |
| Male | 19 | 6 | 28 | 237 | 34 | 86 | 33 | 10 | 453 |
| Female | 2 | 0 | 2 | 19 | 4 | 2 | 1 | 0 | 30 |
| Total | 21 | 6 | 30 | 256 | 38 | 88 | 34 | 10 | 483 |
| **Age group** | Inside the structure where household members sleep | Kitchen (different structure from where household members sleep) | On the veranda | In the courtyard | Storage room | Chicken coop/wooden cage | Other rooms (except storeroom and stable) | Other the wall/Hangar | Total |
| [20-35[ | 4 | 1 | 4 | 32 | 3 | 19 | 4 | 1 | 68 |
| [35-50[ | 6 | 2 | 14 | 99 | 18 | 36 | 11 | 5 | 191 |
| [50-65[ | 5 | 2 | 11 | 81 | 12 | 27 | 13 | 4 | 155 |
| [65 et +[ | 6 | 1 | 1 | 44 | 5 | 6 | 6 | 0 | 69 |
| Total | 21 | 6 | 30 | 256 | 38 | 88 | 34 | 10 | 483 |
| **Education** | Inside the structure where household members sleep | Kitchen (different structure from where household members sleep) | On the veranda | In the courtyard | Storage room | Chicken coop/wooden cage | Other rooms (except storeroom and stable) | Other the wall/Hangar | Total |
| No formal education | 16 | 5 | 18 | 184 | 25 | 57 | 23 | 9 | 337 |
| Formal education | 2 | 0 | 5 | 39 | 8 | 23 | 8 | 0 | 85 |
| Adult literacy | 3 | 1 | 7 | 33 | 5 | 8 | 3 | 1 | 61 |
| Total | 21 | 6 | 30 | 256 | 38 | 88 | 34 | 10 | 483 |
| **Main activity** | Inside the structure where household members sleep | Kitchen (different structure from where household members sleep) | On the veranda | In the courtyard | Storage room | Chicken coop/wooden cage | Other rooms (except storeroom and stable) | Other the wall/Hangar | Total |
| Poultry farming | 1 | 0 | 1 | 26 | 2 | 12 | 21 | 0 | 63 |
| Other livestock farming | 1 | 1 | 2 | 5 | 0 | 5 | 1 | 0 | 15 |
| Crop farming | 19 | 4 | 26 | 220 | 32 | 63 | 11 | 9 | 384 |
| Salaried employment | 0 | 0 | 1 | 2 | 0 | 0 | 0 | 0 | 3 |
| Small trader | 0 | 0 | 0 | 0 | 2 | 4 | 1 | 0 | 7 |
| Gold panning | 0 | 0 | 0 | 2 | 0 | 4 | 0 | 0 | 6 |
| Vegetables production | 0 | 0 | 0 | 0 | 1 | 0 | 0 | 1 | 2 |
| Other | 0 | 1 | 0 | 1 | 1 | 0 | 0 | 0 | 3 |
| Total | 21 | 6 | 30 | 256 | 38 | 88 | 34 | 10 | 483 |
| **Marital status** | Inside the structure where household members sleep | Kitchen (different structure from where household members sleep) | On the veranda | In the courtyard | Storage room | Chicken coop/wooden cage | Other rooms (except storeroom and stable) | Other the wall/Hangar | Total |
| Not married | 0 | 0 | 1 | 4 | 1 | 2 | 0 | 0 | 8 |
| Married monogamous | 9 | 4 | 20 | 144 | 21 | 61 | 23 | 7 | 289 |
| Married polygamous | 10 | 2 | 7 | 91 | 12 | 23 | 10 | 3 | 158 |
| Concubinage | 0 | 0 | 0 | 1 | 0 | 0 | 0 | 0 | 1 |
| Divorced | 0 | 0 | 1 | 1 | 0 | 0 | 0 | 0 | 2 |
| Widow | 2 | 0 | 1 | 15 | 2 | 2 | 1 | 0 | 25 |
| Total | 21 | 6 | 30 | 256 | 38 | 88 | 34 | 10 | 483 |
