# Supplementary material for: Characteristics of chicken production systems in rural Burkina Faso: A focus on One Health related practices and food security
Source: PLoS One. 2025 Feb 3;20(2):e0317898. doi: 10.1371/journal.pone.0317898 (PMC11790147; doi:10.1371/journal.pone.0317898)
Supplement: S7 Table — (DOCX) [file pone.0317898.s007.docx]

Table S7: Suspected diseases that have affected chickens in the past 3 months prior to the survey according to the farmers

| **Age group** | Newcastle | Fowl pox | Both | No disease | Don’t know | Total |
| --- | --- | --- | --- | --- | --- | --- |
| [20-35[ | 43 | 1 | 18 | 4 | 2 | 68 |
| [35-50[ | 114 | 7 | 65 | 5 | 0 | 191 |
| [50-65[ | 91 | 5 | 49 | 7 | 3 | 155 |
| [65 et +[ | 37 | 0 | 25 | 5 | 2 | 69 |
| Total | 285 | 13 | 157 | 21 | 7 | 483 |
| **Education** | Newcastle | Fowl pox | Both | of t | kno | Total |
| No formal education | 189 | 11 | 114 | 17 | 6 | 337 |
| Formal education | 55 | 1 | 26 | 2 | 1 | 85 |
| Adult literacy | 41 | 1 | 17 | 2 | 0 | 61 |
| Total | 285 | 13 | 157 | 21 | 7 | 483 |
| **Main activity** | Newcastle | Fowl pox | Both | of t | kno | Total |
| Poultry farming | 27 | 3 | 31 | 2 | 0 | 63 |
| Other livestock farmi | 13 | 1 | 1 | 0 | 0 | 15 |
| Crop farming | 229 | 8 | 121 | 19 | 7 | 384 |
| Salaried employment | 2 | 0 | 1 | 0 | 0 | 3 |
| Small trader | 6 | 0 | 1 | 0 | 0 | 7 |
| Gold panning | 4 | 1 | 1 | 0 | 0 | 6 |
| Vegetables production | 1 | 0 | 1 | 0 | 0 | 2 |
| Other | 3 | 0 | 0 | 0 | 0 | 3 |
| Total | 285 | 13 | 157 | 21 | 7 | 483 |
| **Marital status** | Newcastle | Fowl pox | Both | of t | kno | Total |
| Not married | 7 | 0 | 0 | 1 | 0 | 8 |
| Married monogamous | 170 | 7 | 96 | 11 | 5 | 289 |
| Married polygamous | 88 | 5 | 55 | 8 | 2 | 158 |
| Concubinage | 0 | 0 | 1 | 0 | 0 | 1 |
| Divorced | 2 | 0 | 0 | 0 | 0 | 2 |
| Widow | 18 | 1 | 5 | 1 | 0 | 25 |
| Total | 285 | 13 | 157 | 21 | 7 | 483 |
